# Molecular Markers Allow to Remove Introgressed Genetic Background: A Simulation Study

**DOI:** 10.1371/journal.pone.0049409

**Published:** 2012-11-09

**Authors:** Carmen Amador, Miguel Ángel Toro, Jesús Fernández

**Affiliations:** 1 INIA, Ctra. De La Coruña Km. 7.5, Madrid, Spain; 2 ETSIA, UPM, Ciudad Universitaria, Madrid, Spain; University of Lausanne, Switzerland

## Abstract

The maintenance of genetically differentiated populations can be important for several reasons (whether for wild species or domestic breeds of economic interest). When those populations are introgressed by foreign individuals, methods to eliminate the exogenous alleles can be implemented to recover the native genetic background. This study used computer simulations to explore the usefulness of several molecular based diagnostic approaches to recover of a native population after suffering an introgression event where some exogenous alleles were admixed for a few generations. To remove the exogenous alleles, different types of molecular markers were used in order to decide which of the available individuals contributed descendants to next generation and their number of offspring. Recovery was most efficient using *diagnostic* markers (i.e., with private alleles) and least efficient when using alleles present in both native and exogenous populations at different frequencies. The increased inbreeding was a side-effect of the management strategy. Both values (% of native alleles and inbreeding) were largely dependent on the amount of exogenous individuals entering the population and the number of generations of admixture that occurred prior to management.

## Introduction

Mixing populations may improve fitness by avoiding inbreeding depression [1], but it can also have negative consequences. One of those is outbreeding depression due to hybridization and break up of co-adapted gene complexes (when local adaptation exists) which can lead to extinction in both wild and domestic populations [2], [3]. Invasive species can also represent a significant and often irreversible threat, affecting human health, economic losses or disruption of ecosystems when autochthonous species are replaced [4]. When dealing with farmed animals, interbreeding can also be undesirable. Several domestic breeds are associated with quality products of economic interest (e.g., *Iberian* pigs, *Reggiana* dairy cows), directly linked with their pure genetic background [5]. Purebreds are also required for activities and competitions or just for aesthetic reasons as for breeds of horses and dogs [6], [7].

Introgression can be particularly risky for endangered populations of wild animals threatened by their domestic relatives. This situation, more frequent than expected, increases because of human-mediated actions [8]. Examples have been documented in many species:: wild and stocked grayling (*Thymallus thymallus*) [9], wild and domestic cats (*Felis silvestris catus* and *F. silvestris* spp.) [10], european mink (*Mustela lutreola*) and polecat (*M. putorius*) [11], wild and domestic American mink (*Neovison vison*) [12], wild and domestic partridges (*Alectoris rufa* and *A. graeca*) [13], and wild and domestic quail (*Coturnix japonica and C. coturnix*) [14]. In these examples, introgression is a threat to the native species, which is vulnerable because it is being replaced. This gene flow can imply that important species in some ecosystems become more endangered, so actions should be taken to preserve them.

In farmed animals, crossbreeding of local breeds with more productive ones has often led to the loss of specific adaptations. This should also be reversed to preserve the particularities of the indigenous breeds which possess gene combinations and special adaptations (e.g., disease resistance, adaptation to harsh conditions) not shown in other breeds [15], [16].

In a previous study [17], different scenarios were simulated to try to cover the complexity of the introgression events, varying the number of foreign individuals entering the population and the number of generations elapsed before recovery management began. In that study, the information of the pedigree (complete since the introgression took place) was used to select which individuals should contribute to the next generation in order to remove non-native alleles. Among the tested methods, minimisation of the coancestry with the foreign founders provided the best results regarding the amount of exogenous genetic material eliminated. This strategy allowed to remove part of the exogenous alleles in most scenarios. However, even small introgression phenomena (i.e., few foreign individuals and few generations of admixture) could lead to irrecoverable situations, encouraging strict control of the populations and rapid action in case of undesired introgression. The study also pointed out the problem of increased inbreeding and coancestry associated with the removal process.

As many studies have shown, molecular markers can help in the detection of hybrids and in the discovery of introgression events [18]. Therefore, it would be expected that they can also be used to accomplish the opposite task (i.e., identifying purer individuals, helping in the removal of exogenous alleles from the population). Thus, the objective of this study was to analyse, through computer simulations, the efficiency of several marker based methods to remove undesired exogenous alleles from a mixed population. The study assumes a tight control on the reproductive process that is only feasible in captive populations. In wild populations, it would require an ex-situ program to control the individuals for the de-introgression process and, afterwards, a reintroduction of the purest individuals into their natural habitats. The methods could be easily applied in farmed animals, as they are already routinely managed. Several scenarios where exogenous genetic information was admixed in a native population were simulated. Then, molecular marker based techniques were used to recover the native background.

## Methods

### Population structure

We considered individuals from two different populations (native and exogenous, harbouring different genetic information). We assume that, at some point, several foreign individuals entered the native population (introgression event), and mated randomly for a variable number of discrete generations (admixture period). After that, ten discrete generations of management were simulated. Base populations with two different sizes, 100 individuals and 20 individuals (50% males and 50% females), were simulated with a constant size and sex ratio along generations. One hundred individuals could represent a typical population size for local breeds of domestic animals. Twenty individuals is a more realistic scenario when dealing with conservation programs of endangered wild species, which usually have smaller population sizes.

Two factors determined the different introgression scenarios.

#### Number of exogenous individuals

In the population with 100 individuals, 10 to 50 exogenous individuals (sexes randomly assigned in each replicate) were included as part of the base population. The remaining individuals, up to 100, were native, to complete the base population, implying an introgression percentage of 10 to 50%. In the population with 20 individuals, the percentage of simulated introgression was the same (10–50%) by including 2, 4, 6, 8 or 10 exogenous individuals in the base population and completing up to 20 with native individuals.

#### Number of generations without management (admixture period)

One to five generations with random contributions and mating were simulated prior to management, to simulate the admixture of the foreign alleles that were included in the base population into the native genetic pool. Each offspring was generated by randomly sampling parents with replacement.

The genome of each individual was made up of one 20 M chromosome, with a total of 2000 multiallelic loci (non-marker loci). Individuals in the base population (all non inbred and unrelated) carried two different alleles at each locus and, thus, were all heterozygous (the number of alleles per locus in generation 0 was 200 or 40, for *N* = 100 or *N* = 20, respectively). Thus, the situation was completely informative regarding the maintenance of diversity. Besides, the origin of each allele (native or exogenous) could be determined, and consequently was useful for the evaluation of the recovery efficiency. These 2000 multiallelic loci were used to evaluate the efficiency of the methods in eliminating exogenous alleles and the consequences on the global genetic diversity. When creating gametes, a Poisson distributed (λ = 20) number of crossing-overs (one crossover is expected on average in each Morgan) with no interference were generated in random positions over the chromosome.

Additionally, markers were simulated (evenly spaced along the genome) to be used in the removal of the foreign alleles. Different situations were considered:

#### Diagnostic markers

Five to 20 biallelic markers were simulated. In the base population, all native individuals were homozygous for *allele 1* in all markers, and all foreigners were homozygous for *allele 2*. Therefore, alleles were private for native or foreign individuals (see Table 1).

**Table 1:** Combinations of frequencies in the native and the exogenous population of each possible allele in each type of marker simulated.

Marker type	Population	*Allele 1*Freq.	*Allele 2*Freq.	*Allele 3*Freq.	*Allele 4*Freq.
*Diagnostic*	*Native*	1	0	—	—
	*Exogenous*	0	1	—	—
*Diagnostic-like*	*Native*	0.80	0.20	—	—
	*Exogenous*	0.20	0.80	—	—
*Extra Diagnostic-like*	*Native*	0.7	0.3	—	—
		0.8	0.2		
		0.9	0.1		
		0.95	0.05		
		0.99	0.01		
	*Exogenous*	0.5	0.5	—	—
*Non-Diagnostic*	*Native*	0.80	0.07	0.06	0.07
	*Exogenous*	0.07	0.80	0.06	0.07

#### Diagnostic-like markers

Five to 20 biallelic markers were simulated. The two alleles in each marker were present in both populations with very different frequencies. In the native population, *allele 1* was present at frequency 0.8 and *allele 2* at frequency 0.2, while in the foreign population, the frequencies for *allele 1* and *2* were 0.2 and 0.8, respectively. This distribution of frequencies was simulated to mimic a scenario where alleles are thought to be private but they are not. To further investigate the consequences of this erroneous assumption, extra simulations were run with different sets of frequencies (see Table 1).

#### Non-Diagnostic

Five to 20 markers with four alleles each were simulated. Frequencies of the alleles in the original native and foreign populations are shown in Table 1 and were assumed to be known without error.

### Management

The populations were managed over 10 generations to recover the native genome.

#### Diagnostic and diagnostic-like markers

In every generation of management individuals with the highest number of *native alleles* were chosen to be parents of the next generation. In the *diagnostic* markers scenarios, *native alleles* were those exclusive of the native population, and in the *diagnostic-like* scenarios, *native alleles* were those with the highest frequency (0.8) in native individuals. Both strategies consider the markers as diagnostic, but in the diagnostic-like scenario the allele is always considered to be native, although it is exogenous in 20% of the cases. In that manner we were able to consider the consequences of assuming a marker is diagnostic when it was not.

Thus, among all individuals available in each generation, only those with the maximum number of native alleles contributed to the next generation (at least one male and one female should be selected to allow the contributions from both sexes to be the same). For example, in the extreme case of just one female with the maximum number of *native alleles*, this female will be the mother of all the offspring. According to this procedure, the number of individuals contributing offspring was not the same in each generation of management. When more than one individual had the same (and maximum) number of *native alleles* (the most likely situation), contributions were randomly allocated to any of the individuals in this subset, so the contributions could be different in each individual.

#### Non-Diagnostic markers

To recover the native background, the contributions to the next generation were decided by minimising the expected genetic distance between the original native population (with frequencies assumed known without error) and the current population. Three genetic distances were considered:

*Cavalli-Sforza and Edwards Chord Distance* [19]

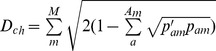



*Nei's Minimum Distance* [20], [21]

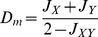
where
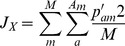


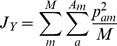


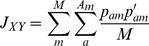



*Kullback-Leibler Divergence* [22]

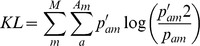



In all cases, *A_m_* is the total number of alleles in locus *m*, *M* is the total number of marker loci, *p_am_* is the frequency of allele *a* at locus *m* in the original native population, and *p′_am_* is the expected frequency in the next generation due to a particular scheme of contributions (i.e., certain number of offspring per parent). This can be calculated as follows:
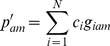
where *N* is the number of individuals, *c_i_* is the relative contribution of individual *i* to the next generation, and *g_i am_* is the probability of gametes from individual *i* carrying allele *a* of marker *m* (1 for homozygotes *aa*, 0.5 for heterozygotes and 0 for individuals not carrying allele *a*).

All the optimizations were solved using *simulated annealing* algorithms [23], [24]. Once contributions were decided, minimum coancestry matings were arranged in all cases using the *Hungarian* algorithm [25]. Twenty replicates per scenario were simulated and results presented are averages across replicates.

The pedigree of the populations was recorded during the two periods (i.e., admixture and management), but never used in the management.

### Measuring the consequences of management

For every generation, the native founder representation was calculated (i.e., the proportion of alleles, calculated from non-marker loci, coming originally from native founders), to evaluate the efficiency of the strategies in the de­introgression task (removal of foreign alleles). Inbreeding coefficient and mean coancestry values were calculated from the pedigree data. Additionally, observed homozygosity was computed from the non-marker multiallelic loci.

## Results

### Native representation

**Figure 1:**
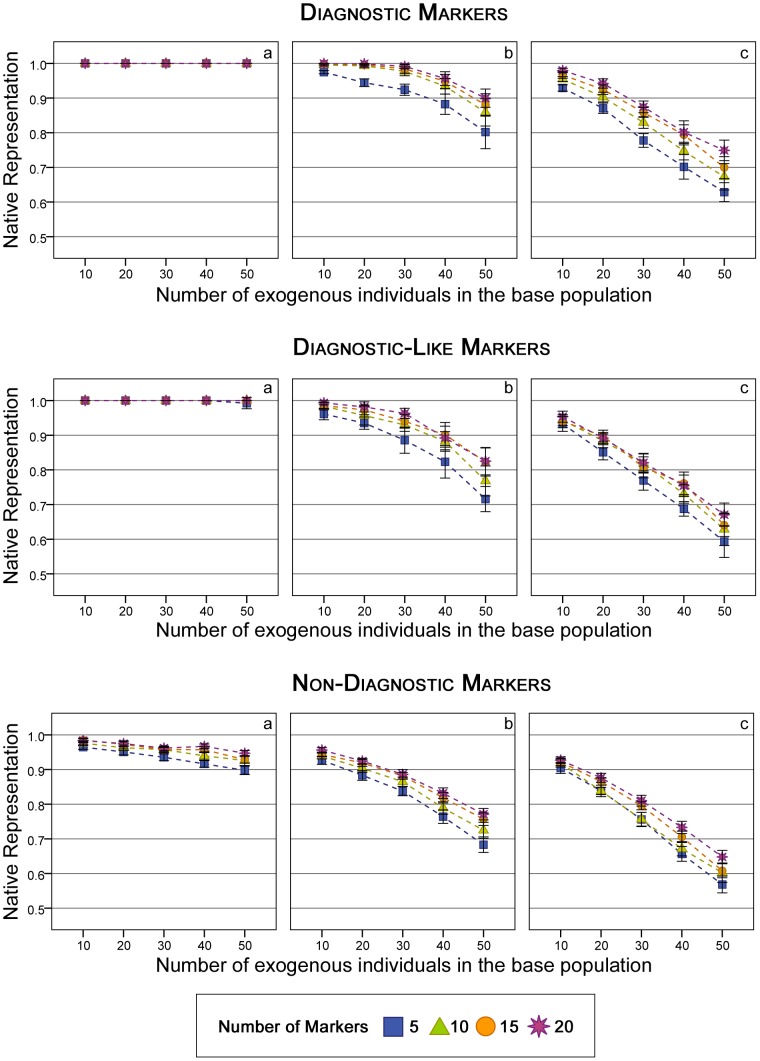
Native representation under the different management strategies (*N* = 100). Values shown are those obtained at the 10^th^ generation of management. Upper panel: *Diagnostic* markers, Medium panel: *Diagnostic-like* markers, Lower panel: *Non-Diagnostic* markers. a) one generation of admixture b) three generations of admixture, c) five generations of admixture. Vertical bars represent the 95% percentiles.

Results for native representation (i.e., the proportion of alleles coming from native founders, *NR*) are shown in Fig. 1 for five to 20 *diagnostic*, *diagnostic-like*, and *non-diagnostic* markers in the population with 100 individuals. As expected, the results obtained with the *diagnostic* markers were the most efficient of the three types. In all cases there was some recovery using *diagnostic* markers, and complete recovery of the native background in cases when the admixture period was short. When using *diagnostic-like* markers the usefulness decreased because of the assumption of them being *diagnostic*, but still, there was some recovery must be noted. The degree of recovery of the native background was always lower than with the *diagnostic* markers, but the differences were small, particularly in scenarios with a long admixture period (5 generations). The influence of the number of markers used in the management is also notable, with higher levels of information (more markers) yielding better results.

More detailed results are shown in Table 2 for 20 *diagnostic-like* markers with several *native allele* frequencies and intermediate frequencies of the alleles in the foreign population (i.e., 0.5/0.5), in scenarios with an admixture period of five generations and after ten generations of management (population *N* = 100). As expected, the greater the frequency of the *native allele*, the higher the removal of undesired introgression as we approached the *diagnostic* scenario, even though the frequency of the *native allele* in the exogenous population was high.

**Table 2:** Results obtained for Native Representation (*NR*) and inbreeding coefficient (*F*) after managing during 10 generations with 20 *diagnostic-like* markers with different *native allele* frequencies (admixture period of 5 generations).

*Native allele* frequency		Num. exogenous individuals				
		10	20	30	40	50
0.70	*NR*	0.929±0.008	0.840±0.012	0.735±0.012	0.649±0.015	0.584±0.019
	*F*	0.417±0.013	0.443±0.009	0.456±0.008	0.462±0.009	0.483±0.010
0.80	*NR*	0.930±0.008	0.866±0.012	0.785±0.019	0.714±0.017	0.588±0.017
	*F*	0.334±0.015	0.365±0.009	0.395±0.009	0.412±0.011	0.466±0.010
0.90	*NR*	0.950±0.007	0.867±0.011	0.792±0.017	0.722±0.017	0.620±0.017
	*F*	0.245±0.012	0.300±0.012	0.345±0.009	0.355±0.014	0.369±0.011
0.95	*NR*	0.952±0.006	0.893±0.011	0.803±0.015	0.732±0.018	0.638±0.016
	*F*	0.208±0.013	0.249±0.011	0.309±0.010	0.336±0.012	0.371±0.012
0.99	*NR*	0.954±0.004	0.903±0.010	0.814±0.012	0.729±0.016	0.640±0.016
	*F*	0.099±0.005	0.191±0.013	0.266±0.009	0.313±0.014	0.346±0.012

In all cases the frequency of the *native allele* in the foreign population was 0.5.

In all cases, the maximum *NR* was reached after three to four generations of management and, in cases with little introgression, the recovery was complete after just one generation of management (data not shown).

The number of exogenous individuals that entered the native population is also a key factor to determine the potential of success, with more exogenous individuals making the situation irrecoverable.

It must be pointed out that in scenarios with a long admixture period, the possibilities of recovery were quite low, even when using information from many markers (see Fig. 1 and Table 2).

Results obtained minimising any of the three genetic distances (using the information of the *non-diagnostic* markers) were similar and, consequently, only the results for the minimisation of the *Kullback-Leibler Divergence* (*KL*) are presented (Fig. 1, lower panel). As in the other scenarios, the ability to recover the original background by minimising *KL* depended on the length of the admixture period. In cases with a short admixture period, a good percentage of native representation could be recovered, even with many introgressed individuals (40–50%). Notwithstanding, in those scenarios with larger admixture periods the restoration was minimal. Again, the recovery increased with the number of markers.

It is remarkable that *diagnostic-like* markers performed better (i.e., leads to higher levels of *NR*) than the *non-diagnostic* markers in all situations, irrespective of the length of the admixture period or the number of exogenous individuals. Results for both types of markers are comparable despite the fact that the number of alleles was not the same, because the frequency of the most common allele was the same in both simulations. Hence, if markers had four alleles at frequencies 0.8/0.07/0.06/0.07 (as in the *non-diagnostic* scenario), managing with the *diagnostic-like* method would imply choosing the *native* allele as the most frequent and taking no action for the rest of alleles, even if there is only one, or three more.

Some other simulations were carried out with different combinations of frequencies and numbers of alleles (data not shown). Results were always linked to the differences in the frequencies between both populations. Cases with a large number of alleles gave lower values of recovery because allele frequencies become more similar between native and foreign populations, making it impossible to discern the origin of alleles.

The results for native representation in the population with 20 individuals (data not shown) had the same pattern as for 100 individuals. The best results were obtained with *diagnostic* markers, with *diagnostic-like* markers performing better than minimising the genetic distances in all scenarios, confirming the ranking in the efficiency of the methods. However, the percentage of native genome recovered in the simulations with 20 individuals was slightly lower in all cases, especially when the percentage of introgression was high (40–50%). Scenarios with an admixture period of five generations were almost irrecoverable (irrespective of the method) pointing out the importance of acting soon, especially for small populations.

### Inbreeding coefficient

**Figure 2:**
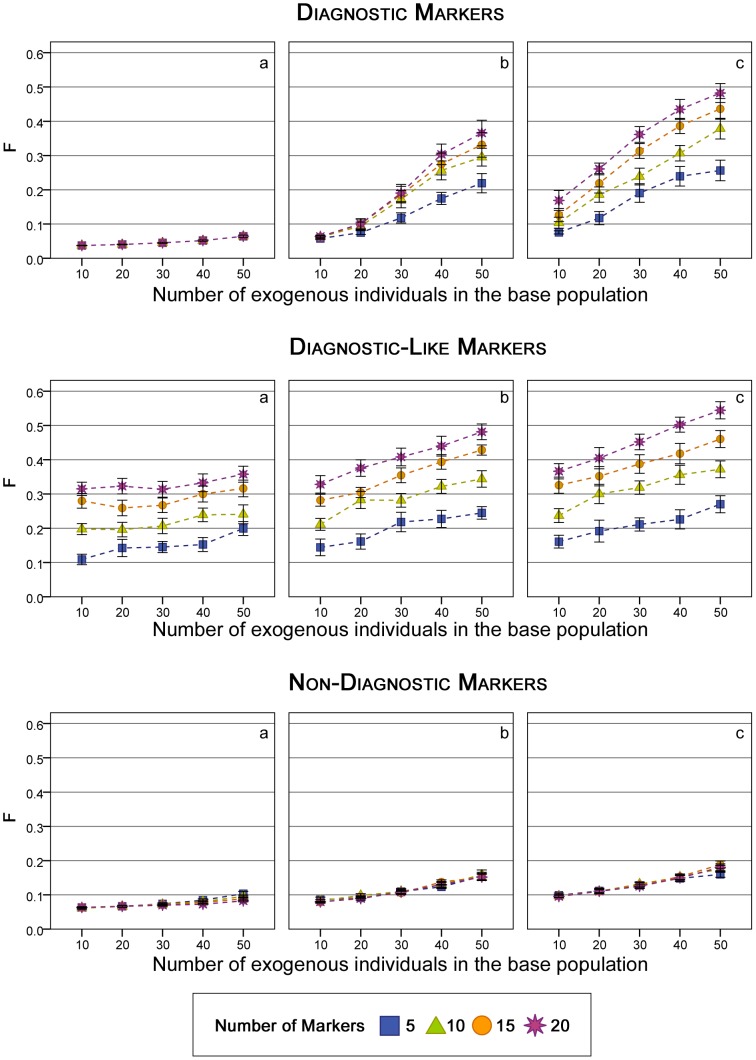
Inbreeding coefficient under the different management strategies (*N* = 100). Values shown are those obtained at the 10^th^ generation of management. Upper panel: *Diagnostic* markers, Medium panel: *Diagnostic-like* markers, Lower panel: *Non-Diagnostic* markers a) one generation of admixture b) three generations of admixture, c) five generations of admixture. Vertical bars represent the 95% percentiles.

Trends for mean coancestry and inbreeding coefficients were similar. Consequently, only the levels of inbreeding (*F*) after ten generations of management are presented in Fig. 2, for five to 20 markers of the three types. The increased inbreeding is a clear side-effect of the de­introgression process when managing with either *diagnostic* or *diagnostic-like* markers. The *F* values were higher in cases with more introgression to remove. This is because the method restricts the number of individuals contributing to the next generation. When the levels of introgression are higher, fewer individuals are expected to be pure, and, thus, selecting fewer individuals to reproduce will raise inbreeding levels more quickly.

When evaluating scenarios with *diagnostic-like* markers, the increase in inbreeding was more pronounced than with the *diagnostic* markers. This was observed for all scenarios (Fig. 2 and Table 2). The increase in inbreeding (as a consequence of the method) was higher with a lower frequency of the *native allele*. This is because the number of contributing individuals was lower than with *diagnostic* management (data not shown). As the private allele frequency was actually lower than in the diagnostic scenario (since fewer individuals carry that allele), fewer individuals will be selected and fewer will contribute offspring, thereby increasing inbreeding. Moreover, these individuals are not the purest, because the alleles are not really private, and the results of the Native Representation are worse, as shown in Figure 1.

As mentioned before, the maximum *NR* was reached after 3 or 4 generations of management when there are no longer differences in *NR* between individuals. Therefore, the method selects contributions randomly in subsequent generations. From that point on, management could be switched to a method devoted exclusively to maintain diversity and avoiding the increase in inbreeding, like minimum coancestry contributions [26].

No significant differences were found between using any of the three genetic distances for de­introgression purposes (nor for inbreeding results either), so only the *KL* results are presented in Fig. 2 (lower panel). The increase in inbreeding due to the ten generations of management was small in all cases. Lower *F* values were obtained when using *non-diagnostic* rather than *diagnostic* or *diagnostic-like* markers.

Inbreeding was higher in the population with 20 individuals than in the *N* = 100 simulations, due to the smaller population size. But as in the previous simulations, the same pattern was found regarding the comparisons between type of markers and removal strategies (data not shown).

To further analyse the consequences of de-introgression over the increase in inbreeding, extra simulations were performed including a restriction in the maximum number of offspring per individual (5 or 10). Obviously, this restriction alleviated the increase in inbreeding of the population, but reduced the power of recovery. Notwithstanding, a significant recovery of the native genome is still observed, as shown in Table 3. When the maximum number of offspring was set to 10, the removal was still quite effective, with an important decrease in inbreeding levels. Cases with a more stringent restriction (maximum of 5 descendants per individual) removed less exogenous alleles but with a smaller decrease in *F*. This implies that each case can be studied individually to remove the exogenous genome while simultaneously controlling for inbreeding, adapted to the particular characteristics of the population.

**Table 3:** Results obtained for Native Representation (*NR*) and inbreeding coefficient (*F*) after managing during 10 generations with 20 markers of each type (*N* = 100, admixture period of 5 generations) under the different inbreeding control strategies (*NR* errors ranging between 0.003 and 0.016, *F* errors between 0.001 and 0.015).

			Num. Exogenous individuals				
Marker type	F control		10	20	30	40	50
*Diagnostic*	*No restriction*	**NR**	0.980	0.942	0.874	0.802	0.749
		**F**	0.169	0.261	0.361	0.435	0.482
	*Max offspring = 10*	**NR**	0.969	0.914	0.836	0.769	0.699
		**F**	0.094	0.122	0.144	0.171	0.181
	*Max offspring = 5*	**NR**	0.958	0.897	0.816	0.728	0.651
		**F**	0.076	0.088	0.097	0.103	0.103
*Diagnostic-like*	*No restriction*	**NR**	0.953	0.892	0.821	0.754	0.671
		**F**	0.366	0.404	0.452	0.502	0.544
	*Max offspring = 10*	**NR**	0.943	0.882	0.801	0.727	0.638
		**F**	0.143	0.155	0.168	0.180	0.187
	*Max offspring = 5*	**NR**	0.934	0.868	0.780	0.687	0.609
		**F**	0.095	0.096	0.101	0.101	0.102
*Non-Diagnostic*	*No restriction*	**NR**	0.927	0.876	0.810	0.733	0.648
		**F**	0.095	0.111	0.125	0.150	0.180
	*Max offspring = 10*	**NR**	0.929	0.877	0.803	0.727	0.643
		**F**	0.091	0.096	0.104	0.117	0.126
	*Max offspring = 5*	**NR**	0.925	0.866	0.792	0.704	0.614
		**F**	0.071	0.073	0.079	0.083	0.086

### Observed Homozygosity

The evolution of the observed homozygosity calculated from the non-marker multiallelic loci (representing pedigree inbreeding at the genomic level) agreed with the results obtained from the inbreeding coefficient in all scenarios (data not shown). As the 2000 loci were completely informative in the base population, observed homozygosity also measured the identity by descent, providing the same results.

## Discussion

When introgression of genetic material into a population is undesirable actions can be taken to recover the original native genetic pool and remove the exogenous alleles. Examples include livestock populations of economic interest, or in natural populations, endangered by the introgression of exogenous invaders. For a successful recovery, all the available information could be useful. Genealogies have provided good results [17] but have several limitations, including the requirement of a perfectly recorded pedigree.

In the absence of a pedigree, molecular markers have been used to detect introgression [18] and to differentiate between native and exogenous origin when dealing with admixed populations [27]. In this paper, we demonstrate that they can also be helpful in removing exogenous alleles. Our results show that using molecular markers works reasonably well and that the efficiency increases with the number of markers, as expected.

The recovery was highest using *diagnostic* markers, because their private alleles can identify clearly native and foreign origins of alleles in the candidate individuals. Despite some examples of private alleles that have been found at the level of species or subspecies [28]–[30] private alleles are normally uncommon in closely related populations [31]. Unfortunately, this is the most likely situation when dealing with introgression.

Our results show that population size has an impact on the recovery of the native genome indicating that the number of individuals in a population is crucial in the de­introgression process. A larger number of individuals increases the power of recovery of these methods since there is a greater probability of finding the purest individuals. In any case, the diagnostic markers were able to remove some exogenous alleles even in the smallest population, except for the most introgressed scenarios (admixture period of 5 generations).

On the other hand, the diagnostic nature of a particular allele may be false and only due to deficient information (e.g., when there are not enough genotyped animals). Results from simulations using *diagnostic-like* markers (i.e., incorrectly assumed to have private alleles) showed that even with this incorrect information, some percentage of native background can be restored. This is true even in the worst case scenario, in which the allele considered to be *native* can be present in the exogenous population at a frequency as high as 0.5 (Table 2). Finding enough markers with relatively extreme frequencies can lead to an acceptable recovery.

When we are aware of markers that are not diagnostic, relying on genetic distances may also help recover much of the original genetic background. The greater the differences between allele frequencies in both populations, the greater the recovery. The kind of markers required to apply the genetic distance strategy are more common; the key factor is for the allele frequencies to be different enough between the two populations. Nevertheless, treating them as *diagnostic* by selecting the presence of the most frequent allele has been proven to be more effective than minimising the *KL* divergence (or any of the other two distances, which yielded equal results). This happened in the two population sizes tested, although the differences between *diagnostic-like* and *non-diagnostic* in the population with 20 individuals was not so high.

Using markers as if they were diagnostic (as in the *diagnostic-like* approach) instead of using genetic distances also has another advantage. While genetic distances require good estimates of frequencies in *pure* populations, as well as being sure that the population used as a reference has the same genetic origin as that in the process of de­introgression, selecting individuals based on the presence of an allele requires no assumptions about frequencies.

In addition to the amount of information provided by the markers, the probability of success in de­introgression is clearly related to the percentage of undesired background introgressed in the population and to the length of the admixture period.

The values of *NR* obtained when using pedigree information to recover the native background [17] were very similar to those achieved with the *diagnostic* markers and slightly higher than managing with *diagnostic-like* and *non-diagnostic*. Therefore, a reasonable number of informative markers are enough to achieve the same recovery without requiring the complete genealogy, which is often not available.

Some extra simulations were carried out to test the efficiency of the methods in a smaller genome (number of crossovers simulated through a Poisson distribution with λ = 1, representing one chromosome of 1 M). The efficiency of the methods increased (Fig. S1) because the markers are now more informative, due to the higher linkage disequilibrium between markers and the rest of loci. The *diagnostic* markers are still the best approach to remove the exogenous alleles, but the other two methods allow to recover almost 100% of the native genome in the scenarios tested. Notwithstanding, that genome length is very unrealistic for species in conservation programs.

As with management based on pedigree [17], the de­introgression process using *diagnostic* and *diagnostic-like* markers implies an increase of inbreeding due to the inherent reduction in the number of contributing individuals, in both genome sizes (Fig. S2). The theoretical effective size (*N_e_*, calculated through increase of inbreeding) in each generation of management varied throughout the generations, being small at the beginning of the management (*N_e_* = 3–20 in the *diagnostic*, *N_e_* = 2–15 in the *diagnostic-like* markers, *N_e_* = 30–50 in the *non-diagnostic* markers) and increasing (around 100) when the methods stopped working. It is not realistic to manage a population with one female and one male, and, of course, some kind of control on the loss of diversity should be incorporated. However the restriction on the inbreeding rate (or assuring a minimum number of individuals contributing to each generation) cannot be generalised, because the particular value will depend on the characteristics of the species and the genetic structure of the population.

This side effect of the methods must be taken into account when planning the management by deciding what rate of inbreeding (*ΔF*) we are willing to accept in the process of recovery. As shown in Table 3, explicit restrictions on a maximum offspring per parent, which affects the minimum number of contributing parents, may be implemented to avoid an excessively rapid increase of *F*. Additionally, the number of generations used should be limited to replace the management for de­introgresion with the classical strategy to control the increase in inbreeding. Lengthening the period of removal may lead to little extra recovery with a large rate of *F*, which may be unacceptable. After the maximum *NR* has been reached, the goal of management should be to maximise diversity. However, as it is not possible to predict when maximum *NR* is achieved, it could be advisable to include the restriction on *ΔF* as soon as management begins.

Whereas increasing the number of markers leads to a slight improvement in the recovery of the native background, it also increases inbreeding, particularly when using *diagnostic* and *diagnostic-like* markers. The *F* values were high after managing with a large number of these markers, suggesting another variable to take into account in each situation to get the highest recovery, while losing the least amount of genetic diversity.

On the other hand, the inbreeding obtained by minimizing the *KL* divergence was lower than with the other methods (irrespective of the number of markers), which implies that the number of individuals contributing descendants to the next generation is higher. This must also be taken into account, especially when dealing with populations where *F* needs to be controlled.

The *F* values obtained in the pedigree management [17] were lower than those obtained with the *diagnostic* and *diagnostic-like* markers, excluding cases with a small number of markers and particularly cases with a lot of introgression.

As mentioned above, the acceptable value of *ΔF* depends on the situation and the information available must be assessed before starting the process. In any case, we must be aware that control over inbreeding during management will always imply a loss of efficiency as shown in Table 3. It should be analysed every time, deciding how many exogenous alleles and how much increase of inbreeding is acceptable in each case.

Our results apply only to captive populations, where reproductive control is high. To de­introgress a wild population, a good alternative would be to establish an *ex-situ* population, where the proposed methods are to be applied, providing purer individuals to be released into the natural population.

The conclusion from the present study is that a relatively small number of markers can provide a good tool to remove undesired introgression from a population. The use of this information can lead to a substantial recovery, especially when the presence of *diagnostic* markers or alleles is higher in the population of interest than in the exogenous one. The importance of acting soon to avoid irrecoverable introgression of the exogenous genome is a main concern common to all methods (as in the pedigree approach) and it highlights the importance of prevention to control these populations as much as possible.

## Supporting Information

Figure S1 **Native representation under the different management strategies in the 1 Morgan scenarios (** ***N*** **= 100).** Values shown are those obtained at the 10^th^ generation of management. Upper panel: *Diagnostic* markers, Medium panel: *Diagnostic-like* markers, Lower panel: *Non-Diagnostic* markers. a) one generation of admixture b) three generations of admixture, c) five generations of admixture. Vertical bars represent the 95% percentiles.
(TIF)

Figure S2 **Inbreeding coefficient under the different management strategies in the 1 Morgan scenarios (** ***N*** **= 100).** Values shown are those obtained at the 10^th^ generation of management. Upper panel: *Diagnostic* markers, Medium panel: *Diagnostic-like* markers, Lower panel: *Non-Diagnostic* markers. a) one generation of admixture b) three generations of admixture, c) five generations of admixture. Vertical bars represent the 95% percentiles.
(TIF)

## References

[1] Frankham R, Ballou JD, Briscoe DA (2002) Introduction to Conservation Genetics. Cambridge: Cambridge University Press. 617 p.

[2] Rhymer JM, Simberloff D (1996) Extinction by hybridization and introgression. Annu Rev Ecol Syst 27: 83–109.

[3] Allendorf FW, Leary RF, Spruell P, Wenburg JK (2001) The problems with hybrids: setting conservation guidelines. Trends Ecol Evol 16: 613–622.

[4] Vitousek PM, Mooney HA, Lubchenco J, Melillo JM (1997) Human domination of Earth's ecosystems. Science 277: 494–499.

[5] Dalvit C, De Marchi M, Cassandro M (2007) Genetic traceability of livestock products: A review. Meat Sci 77: 437–449. 22061927 10.1016/j.meatsci.2007.05.027

[6] Parker HG, Kim LV, Sutter NB, Carlson S, Lorentzen TD, et al (2004) Genetic structure of the purebred domestic dog. Science 304: 1160–1164. 15155949 10.1126/science.1097406

[7] MAPA (2003) Estudio y caracterización del sector equino en España. pp. (Ministerio de Agricultura, Pesca y Alimentación).

[8] Simberloff D (1996) Hybridization between native and introduced wildlife species: importance for conservation. Wildlife Biol 2: 143–150.

[9] Sušnik S, Berrebi P, Dovc P, Hansen MM, Snoj A (2004) Genetic introgression between wild and stocked salmonids and the prospects for using molecular markers in population rehabilitation: the case of the Adriatic grayling (*Thymallus thymallus* L. 1785). Heredity 93: 273–282. 15241455 10.1038/sj.hdy.6800500

[10] Beaumont M, Barratt EM, Gottelli D, Kitchener AC, Daniels MJ, et al (2001) Genetic diversity and introgression in the Scottish wildcat. Mol Ecol 10: 319–336. 11298948 10.1046/j.1365-294x.2001.01196.x

[11] Cabria MT, Michaux JR, Gómez-Moliner BJ, Skumatov D, Maran T, et al (2011) Bayesian analysis of hybridization and introgression between the endangered european mink (*Mustela lutreola*) and the polecat (*Mustela putorius*). Mol Ecol 20: 1176–1190. 21244536 10.1111/j.1365-294X.2010.04988.x

[12] Kidd AG, Bowman J, Lesbarreres D, Schulte-Hostedde AI (2009) Hybridization between escaped domestic and wild American mink (*Neovison vison*). Mol Ecol 18: 1175–1186. 19243512 10.1111/j.1365-294X.2009.04100.x

[13] Negro JJ, Torres MJ, Godoy JA (2001) RAPD analysis for detection and eradication of hybrid partridges (*Alectoris rufa*×*A.graeca*) in Spain. Biol Conserv 98: 19–24.

[14] Barilani M, Deregnaucourt S, Gallego S, Galli L, Mucci N, et al (2005) Detecting hybridization in wild (*Coturnix c. coturnix*) and domesticated (*Coturnix c. japonica*) quail populations. Biol Conserv 126: 445–455.

[15] Taberlet P, Valentini A, Rezaei HR, Naderi S, Pompanon F, et al (2008) Are cattle, sheep, and goats endangered species? Mol Ecol 17: 275–284. 17927711 10.1111/j.1365-294X.2007.03475.x

[16] Maudet C, Luikart G, Taberlet P (2002) Genetic diversity and assignment tests among seven French cattle breeds based on microsatellite DNA analysis. J Anim Sci 80: 942–950. 12002331 10.2527/2002.804942x

[17] Amador C, Toro MA, Fernández J (2011) Removing exogenous information using pedigree data. Conserv Genet 12: 1565–1573.

[18] Groeneveld LF, Lenstra JA, Eding H, Toro MA, Scherf B, et al (2010) Genetic diversity in farm animals – a review. Anim Genet 41: 6–31. 20500753 10.1111/j.1365-2052.2010.02038.x

[19] Cavalli-Sforza LL, Edwards AWF (1967) Phylogenetic Analysis Models and Estimation Procedures. Am J Hum Genet 19: 233–257. 6026583 PMC1706274

[20] Nei M (1987) Molecular evolutionary genetics. New York: Columbia. University Press.

[21] Nei M (1973) Analysis of gene diversity in subdivided populations. P Natl Acad Sci USA 70: 3321–3323. 10.1073/pnas.70.12.3321 PMC427228 4519626

[22] Kullback S (1997) Information theory and statistics. Dover, New York: Courier Dover Publications.

[23] Kirkpatrick S, Gelatt CD, Vecchi MP (1983) Optimization by Simulated Annealing. Science 220: 671–680. 17813860 10.1126/science.220.4598.671

[24] Fernández J, Toro MA (1999) The use of mathematical programming to control inbreeding in selection schemes. J Anim Breed Genet 116: 447–466.

[25] Dantzig GB (1963) Linear programming and extensions. Princeton: Princeton University Press.

[26] Caballero A, Toro MA (2000) Interrelations between effective population size and other pedigree tools for the management of conserved populations. Genet Res 75: 331–343. 10893869 10.1017/s0016672399004449

[27] Oliveira R, Godinho R, Randi E, Ferrand N, Alves PC (2008) Molecular analysis of hybridisation between wild and domestic cats (*Felis silvestris*) in Portugal: implications for conservation. Conserv Genet 9: 1–11.

[28] Roy MS, Geffen E, Smith D, Ostrander EA, Wayne RK (1994) Patterns of differentiation and hybridization in North American wolflike canids, revealed by analysis of microsatellite loci. Mol Biol Evol 11: 553–570. 8078397 10.1093/oxfordjournals.molbev.a040137

[29] Goodman SJ, Barton NH, Swanson G, Abernethy K, Pemberton JM (1999) Introgression through rare hybridization: A genetic study of a hybrid zone between red and sika deer (genus *Cervus*) in Argyll, Scotland. Genetics 152: 355–371. 10224266 10.1093/genetics/152.1.355 PMC1460577

[30] MacHugh DE, Shriver MD, Loftus RT, Cunningham P, Bradley DG (1997) Microsatellite DNA variation and the evolution, domestication and phylogeography of taurine and Zebu cattle (*Bos taurus* and *Bos indicus*). Genetics 146: 1071–1086. 9215909 10.1093/genetics/146.3.1071 PMC1208036

[31] Vilà C, Walker C, Sundqvist AK, Flagstad O, Andersone Z, et al (2003) Combined use of maternal, paternal and bi-parental genetic markers for the identification of wolf–dog hybrids. Heredity 90: 17–24. 12522421 10.1038/sj.hdy.6800175

