# Supplementary figures and images for: Molecular Markers Allow to Remove Introgressed Genetic Background: A Simulation Study
Source: PLoS One. 2012 Nov 9;7(11):e49409. doi: 10.1371/journal.pone.0049409 (PMC3494676; doi:10.1371/journal.pone.0049409)

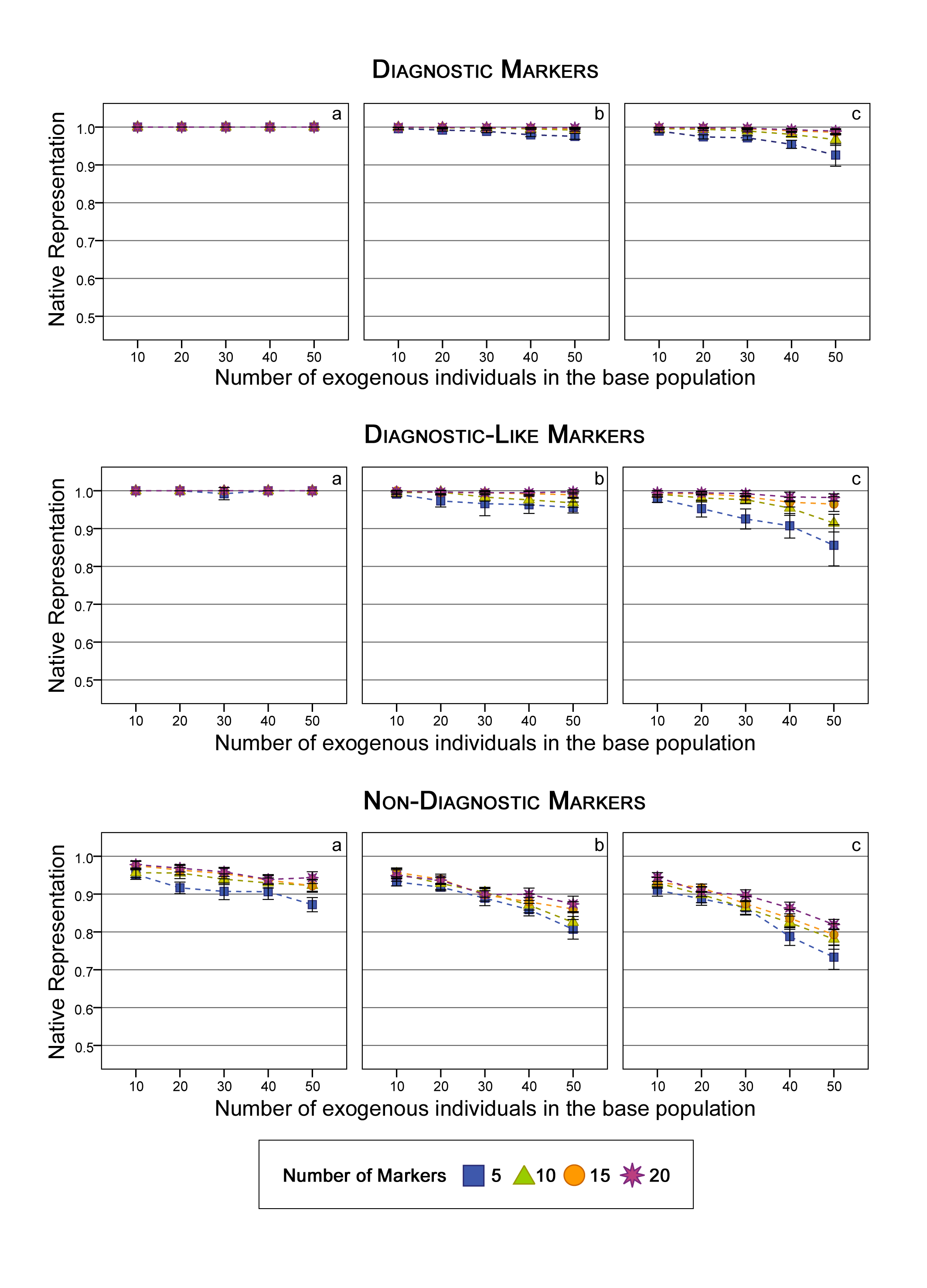

Supplement: Figure S1 — Native representation under the different management strategies in the 1 Morgan scenarios ( N = 100). Values shown are those obtained at the 10th generation of management. Upper panel: Diagnostic markers, Medium panel: Diagnostic-like markers, Lower panel: Non-Diagnostic markers. a) one generation of admixture b) three generations of admixture, c) five generations of admixture. Vertical bars represent the 95% percentiles. (TIF) [file pone.0049409.s001.tif]

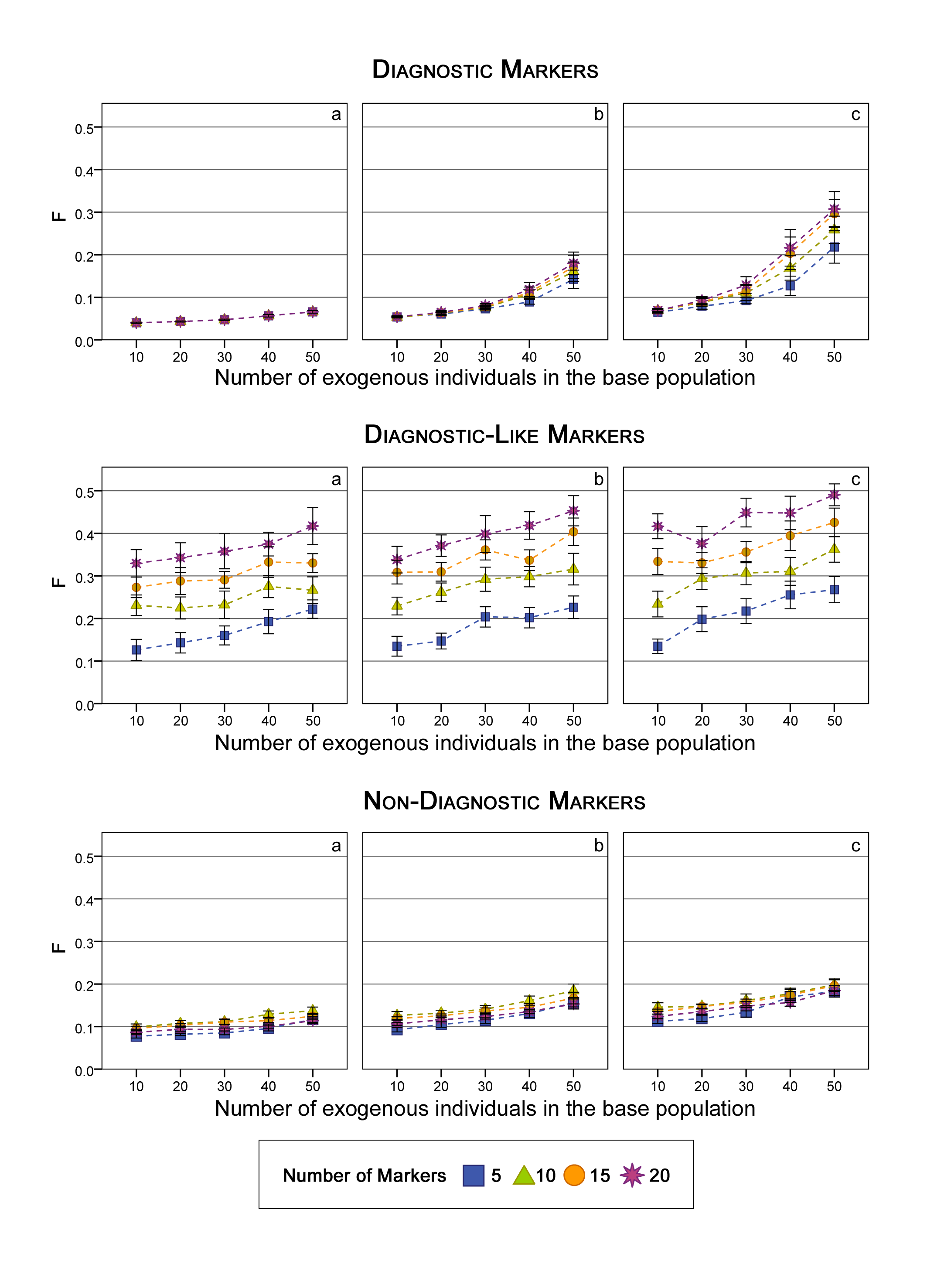

Supplement: Figure S2 — Inbreeding coefficient under the different management strategies in the 1 Morgan scenarios ( N = 100). Values shown are those obtained at the 10th generation of management. Upper panel: Diagnostic markers, Medium panel: Diagnostic-like markers, Lower panel: Non-Diagnostic markers. a) one generation of admixture b) three generations of admixture, c) five generations of admixture. Vertical bars represent the 95% percentiles. (TIF) [file pone.0049409.s002.tif]
